# Ontogenetic Change in the Venom of Mexican Black-Tailed Rattlesnakes (*Crotalus molossus nigrescens*)

**DOI:** 10.3390/toxins10120501

**Published:** 2018-12-01

**Authors:** Miguel Borja, Edgar Neri-Castro, Rebeca Pérez-Morales, Jason L. Strickland, Roberto Ponce-López, Christopher L. Parkinson, Jorge Espinosa-Fematt, Jorge Sáenz-Mata, Esau Flores-Martínez, Alejandro Alagón, Gamaliel Castañeda-Gaytán

**Affiliations:** 1 Facultad de Ciencias Biológicas, Universidad Juárez del Estado de Durango, Av. Universidad s/n. Fracc. Filadelfia, C.P. 35010 Gómez Palacio, Dgo., Mexico; alessandro_53@hotmail.com (M.B.); jsaenz_mata@ujed.mx (J.S.-M.); pollomtz97@hotmail.com (E.F.-M.); 2 Facultad de Ciencias Químicas, Universidad Juárez del Estado de Durango, Av. Artículo 123 s/n. Fracc. Filadelfia, Apartado Postal No. 51, C.P. 35010 Gómez Palacio, Dgo., Mexico; rebecapms@ujed.mx; 3 Instituto de Biotecnología, Universidad Nacional Autónoma de México, Avenida Universidad 2001, Chamilpa, C.P. 62210 Cuernavaca, Mor., Mexico; neri@ibt.unam.mx (E.N.-C.); joserobertoponcelopez21@gmail.com (R.P.-L.); alagon@ibt.unam.mx (A.A.); 4 Programa de Doctorado en Ciencias Biomédicas UNAM, C.P. 04510 México D.F., Mexico; 5 Department of Biological Sciences, Clemson University, 190 Collings St., Clemson, SC 29634, USA; jlstrck@clemson.edu (J.L.S.); viper@clemson.edu (C.L.P.); 6 Department of Forestry and Environmental Conservation, Clemson University, 190 Collings St., Clemson, SC 29634, USA; 7 Facultad de Ciencias de la Salud, Universidad Juárez del Estado de Durango, Calz. Palmas 1, Revolución, 35050 Gómez Palacio, Dgo., Mexico; dr.jorge.espinosa@gmail.com

**Keywords:** antivenom, crotamine-like myotoxins, life history, phenotypic variability, sexual maturity, snake venom metalloproteinases, snake venom serine proteinases

## Abstract

Ontogenetic changes in venom composition have important ecological implications due the relevance of venom in prey acquisition and defense. Additionally, intraspecific venom variation has direct medical consequences for the treatment of snakebite. However, ontogenetic changes are not well documented in most species. The Mexican Black-tailed Rattlesnake (*Crotalus molossus nigrescens*) is large-bodied and broadly distributed in Mexico. To document venom variation and test for ontogenetic changes in venom composition, we obtained venom samples from twenty-seven *C. m. nigrescens* with different total body lengths (TBL) from eight states in Mexico. The primary components in the venom were detected by reverse-phase HPLC, western blot, and mass spectrometry. In addition, we evaluated the biochemical (proteolytic, coagulant and fibrinogenolytic activities) and biological (LD_50_ and hemorrhagic activity) activities of the venoms. Finally, we tested for recognition and neutralization of Mexican antivenoms against venoms of juvenile and adult snakes. We detected clear ontogenetic venom variation in *C. m. nigrescens*. Venoms from younger snakes contained more crotamine-like myotoxins and snake venom serine proteinases than venoms from older snakes; however, an increase of snake venom metalloproteinases was detected in venoms of larger snakes. Venoms from juvenile snakes were, in general, more toxic and procoagulant than venoms from adults; however, adult venoms were more proteolytic. Most of the venoms analyzed were hemorrhagic. Importantly, Mexican antivenoms had difficulties recognizing low molecular mass proteins (<12 kDa) of venoms from both juvenile and adult snakes. The antivenoms did not neutralize the crotamine effect caused by the venom of juveniles. Thus, we suggest that Mexican antivenoms would have difficulty neutralizing some human envenomations and, therefore, it may be necessary improve the immunization mixture in Mexican antivenoms to account for low molecular mass proteins, like myotoxins.

## 1. Introduction

Phenotypic variation as a result of ontogenetic changes during the life of an organism result from differential selection pressures through time [1]. Because of the different pressures, the realized niche also differs through time based on size and maturity. Differences can include the utilization of different habitats [2], risk to predation [3], and diet breadth [2,4,5]. For example, many species of snakes are known to change the prey composition of their diet based on their size [6,7,8,9]. The change in diet is possibly due to the increase in gape size through time [10]. For venomous snakes, there is evidence that many species undergo ontogenetic changes in venom composition that likely accompany the changes in diet [11,12,13,14]. The switch in phenotype tends to be associated with size at sexual maturity when the animals become capable of reproduction. For example, Margres et al. [15] reported that ontogenetic shift in venom composition of *C. adamanteus* occurred at 102 cm, the size at which the species reaches sexual maturity. Phenotypic changes through time have relevant ecological implications for the species as well as medical implications. Understanding ontogenetic venom variation is important from medical and biotechnological perspective because these phenotypic differences have direct consequences for the treatment of snake bites.

In general, more toxic and procoagulant venoms have been detected in newborn or juvenile snakes compared to adults [16,17,18,19,20,21,22]. These ontogenetic differences in biological activities of venoms could be reflected in different clinical manifestations in people bitten by newborn, juvenile or adult snakes. Differences in venom composition during different life-stages may cause antivenoms that are effective against adult venoms to be ineffective against the venom of juveniles [17,23]. Disentangling phenotypic differences due to ontogeny is challenging because many venom phenotypes, such as rattlesnakes, also have a high level of intraspecific and interspecific variability. Some rattlesnake species display different phenotype of venom (e.g., neurotoxic and hemorrhagic) though their distribution, where more severe envenomations may be expected in areas where rattlesnakes with neurotoxic venoms are distributed [24,25]. But, even when the differences are not as diverse as neurotoxic and hemorrhagic, there is still high levels of variability across the geographic distribution of many species [14,21,26].

Rattlesnake (Viperidae: *Crotalus* and *Sistrurus*) venoms are complex cocktails of proteins containing from 30 to over 100 toxins [27] with key ecological functions for prey acquisition and defense [28]. These proteins are grouped into families according to their sequence similarity, activity and structure [29]. The number and proportion of these protein families in snake venoms can vary at inter and intra-specific levels. Generally, the most abundant venom components in rattlesnakes are myotoxins (Myo), phospholipase A_2_s (PLA_2_s), snake venom metalloproteinases (SVMPs), and snake venom serine proteinases (SVSPs) [30]. Intraspecifically, venom variation has been observed in individuals from different geographic regions [31,32,33,34,35], age [13,14,19,21,36,37] and less commonly sex [38,39]. Snake venom variation may be mediated by genetic polymorphisms [40,41], presence/absence of genes that code for specific toxins (e.g., PLA_2_s and SVMPs) [42,43], and/or post-translational modifications [44,45]. Additionally, age-related venom variation has been associated more frequently to differences in the expression of some specific toxin families [15].

There are approximately 50 species of rattlesnakes which are only found in the New World. Mexico contains the highest number of rattlesnake species with 43 (42 *Crotalus* and 1 *Sistrurus*: Data taken from www.reptiledatabase.org). Recent studies have demonstrated important intraspecific venom variation in several rattlesnake species distributed in Mexico including: *Crotalus morulus* [46], *C. culminatus*, *C. simus*, *C. tzabcan* [47], *C. s. scutulatus* [33,48] and *C. polystictus* [21]. Additionally, in several species of rattlesnakes, ontogenetic changes have been documented that correspond with different toxin families. In species such as *C. culminatus* and *C. tzabcan*, a reduction in the amount of myotoxins in venoms as snakes increase their size has been detected [49], but in other species, the opposite occurs [50]. Additionally, SVMPs have been shown to increase as species such as *C. oreganus helleri*, *C. o. oreganus*, *Crotalus polystictus* and *C. simus* get larger [19,21,23,49]. However, even in relatively abundant and widely distributed rattlesnake species, there is little information on the composition of their venoms and the implications of possible ontogenetic venom variation in public health or in the efficacy of antivenoms.

The Black-tailed Rattlesnake (*Crotalus molossus*) is a large bodied species that reaches adult size at approximately 57.6 and 65.3 cm snout-vent length (SVLs), for males and females, respectively [51] Due to the large size differences from neonate (total body length (TBL): ~30 cm) to adult (up to 152.4 cm TBL) [52,53], this species would likely undergo changes in diet composition through time which may be accompanied by venom differences. Balderas-Valdivia et al. [54] reported that *C. m. nigrescens* adults from La Reserva Ecologica del Pedregal in Mexico City feed predominately on rodents, while younger snakes consume predominately lizards. A similar pattern was reported by Werler and Dixon [55] for the closely related Eastern Black-tailed Rattlesnake, *C. ornatus*, from Texas, which shifts from lizards to rodents as they transition from juveniles to adults.

The Mexican Black-tailed Rattlesnake (*Crotalus molossus nigrescens*) is one of four subspecies within *C. molossus* (the other three are *C. m. estebanensis*, *C. m. molossus* and *C. m. oaxacus*) and has the largest distribution in Mexico. Its distribution extends from Northern Durango, Southern Chihuahua and Northern Nuevo León south to Western Veracruz and Northern Oaxaca [53]. Despite the fact that this subspecies is broadly distributed in Mexico, little is known about the biochemical and biological activities of its venom. Previous research reported that *C. m. nigrescens* venom is rich in hydrolytic enzymes such as thrombin-like proteases (21.4 kDa) and proteinase E (75 kDa) [56] and it can have myotoxins α (crotamine-like) [57]. Additionally, ontogenetic venom variation in fibrinolytic and complement inactivation activities was detected in the Northern Black-tailed Rattlesnake (*C. m. molossus*) [58] so it is possible this change also occurs in *C. m. nigrescens*. Although there is no information available about human envenomations by *C. m. nigrescens*, it has been reported that *C. m. molossus* bites induce edema, ecchymosis, coagulopathy, thrombocytopenia, swelling, and hemorrhagic blebs [59,60]. Additionally, compartment syndrome was reported for one patient bitten by this subspecies [61].

Here, we use range-wide sampling of *C. m. nigrescens* to assess venom variability to test for ontogenetic changes in the biochemical and biologic activities of the venom. We were able to collect a range of sizes for this species and place the variability in a geographic context. This study represents the most complete characterization of the biochemical and biological properties of this subspecies and will be of help for future analyses on related subspecies and species.

## 2. Results

### 2.1. Crotalus Molossus Nigrescens Samples

**Figure 1:**
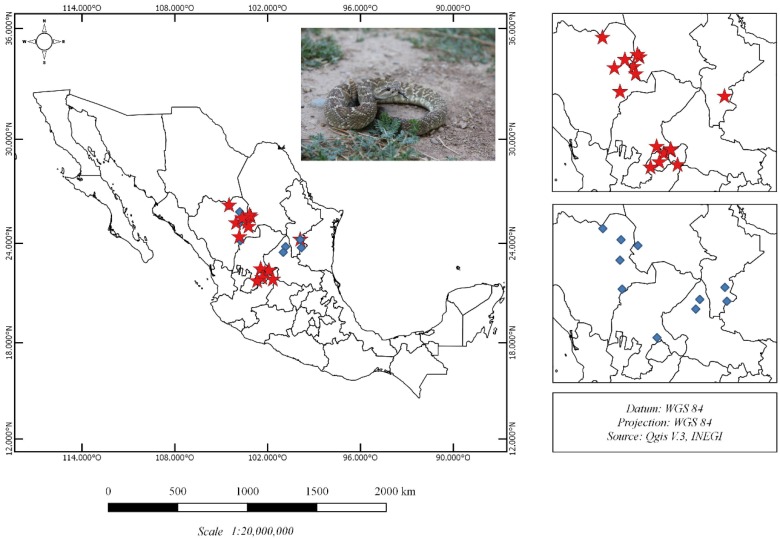
Geographic distribution of the twenty-seven *C. m. nigrescens* samples analyzed in this study. Snakes with a total body length (TBL) less than and greater than 70 cm are represented with blue diamonds and red stars, respectively.

Venom samples were obtained from twenty-seven *C. m. nigrescens* from eight states in Mexico (Figure 1; Table 1). Sixteen snakes were male, nine were females, and two were unknown (CM10 and CM28). The total body length (TBL) of the snakes ranged from 37 to 105 cm (Table 1), but could not be recorded for three individuals (CM21, CM26 and CM28). These three individuals were medium to large adults.

### 2.2. SDS-PAGE

**Figure 2:**
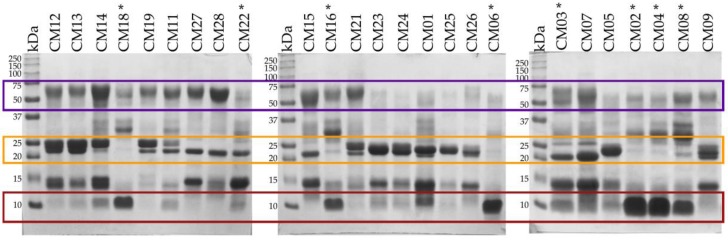
15% SDS-PAGE of *C. m. nigrescens* venoms from Mexico under reducing conditions. Venoms of individuals with a TBL of less than 70 cm are marked with an asterisk (*). Variation in the bands with molecular masses ~10 kDa, from 20 kDa to 25 kD, and from 50 kDa to 75 kDa are marked with red, yellow and purple boxes, respectively. Based on western blot and ESI-MS analyses, these bands correspond to crotamine like-myotoxins, PI-SVMPs, and PIII-SVMPs, respectively. 15 µg of venom was loaded per lane and gels were stained with Coomassie blue. kDa: kilodaltons.

In general, *C. m. nigrescens* venoms had diverse electrophoretic pattern in terms of number and intensity of bands (Figure 2). Venoms from six individuals (CM02, CM04, CM06, CM08, CM16, and CM18) displayed a prominent band near 10 kDa that was absent or less intense in the remaining venoms. These six venoms belong to individuals with a TBL of less than 70 cm. These six venoms also lacked a series of bands located between 20 and 25 kDa (PI-SVMPs) that were detected in the remaining venoms. Other prominent differences were observed in the area located between 50 and 75 kDa (PIII-SVMPs), where 13 of the 27 individuals (CM03, CM07, CM11, CM12, CM13, CM14, CM15, CM16, CM18, CM198, CM21, CM27, and CM28) had a conspicuous band that was less intense in the remaining venoms (Figure 2).

### 2.3. Determination of the Primary Components in C. m. nigrescens Venoms

**Figure 3:**
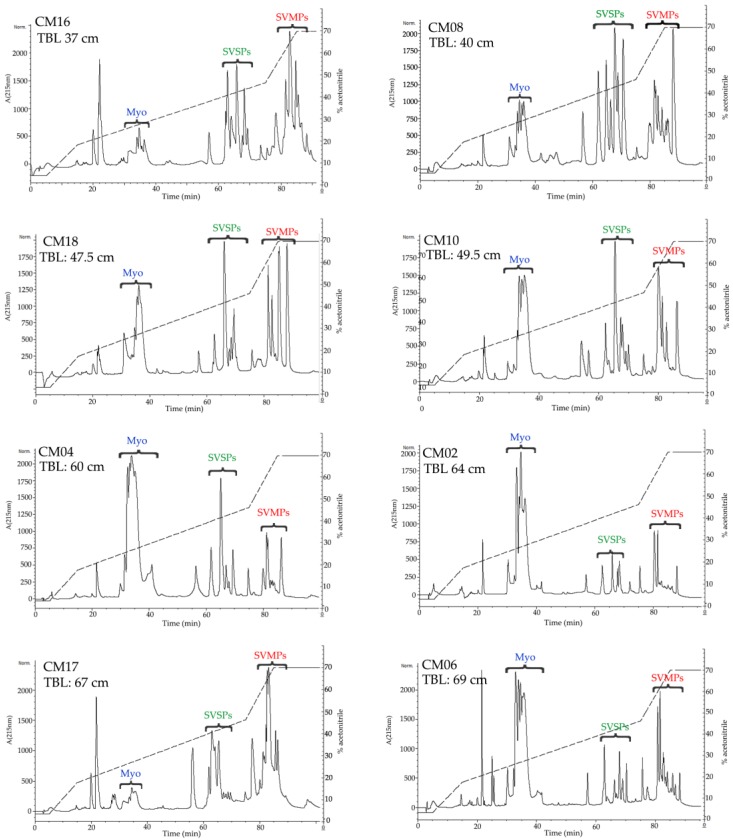
Reverse-phase HPLC chromatograms of representative *C. m. nigrescens* venoms of snakes less than 70 cm ordered by total body length (TBL). An analytic C18 reverse-phase column (Vydac^®^, Deerfield, IL, USA, 218 TP 4.6 mm × 250 mm) was used. Retention time is along the x axis for each panel and labeled every twenty min. Proteins were detected at 215 nm and absorbance is indicated on the left axis. The acetonitrile gradient is shown in the HPLC graph and the percentage value corresponds to the right axis for each panel. Crotamine-like myotoxins (Myo), snake venom serine proteases (SVSPs), and snake venom metalloproteinases (SVMPs) are illustrated in blue, green and red, respectively.

**Figure 4:**
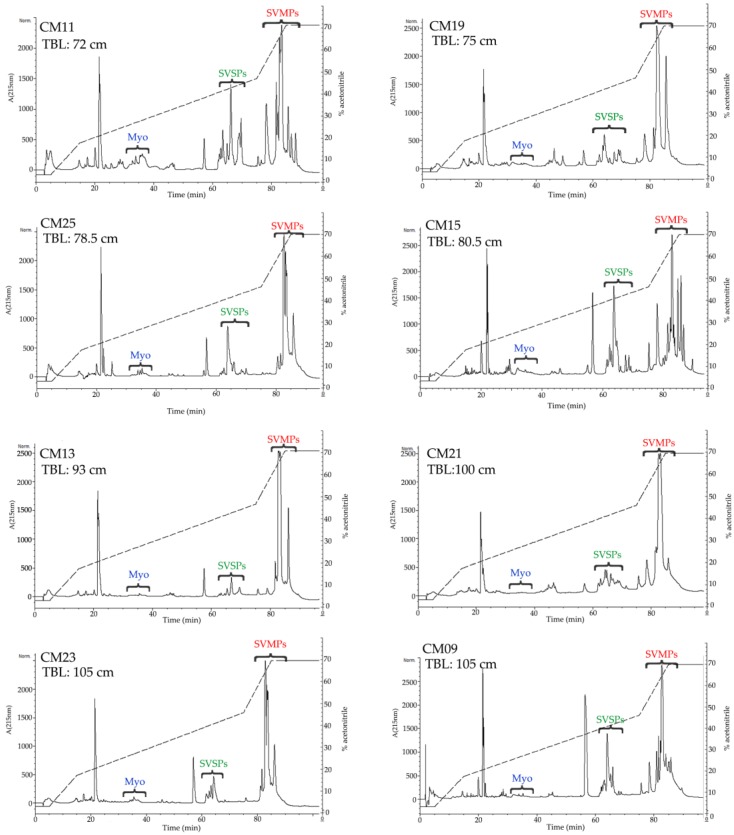
Reverse-phase HPLC chromatograms of representative *C. m. nigrescens* venoms of snakes greater than 70 cm ordered by total body length (TBL). An analytic C18 reverse-phase column (Vydac^®^, Deerfield, IL, USA, 218 TP 4.6 mm × 250 mm) was used. Retention time is along the x axis for each panel and labeled every twenty min. Proteins were detected at 215 nm and absorbance is indicated on the left axis. The acetonitrile gradient is shown in the HPLC graph and the percentage value corresponds to the right axis for each panel. The regions where crotamine-like myotoxins (Myo), snake venom serine proteases (SVSPs), and snake venom metalloproteinases (SVMPs) elute are illustrated in blue, green and red, respectively.

**Figure 5:**
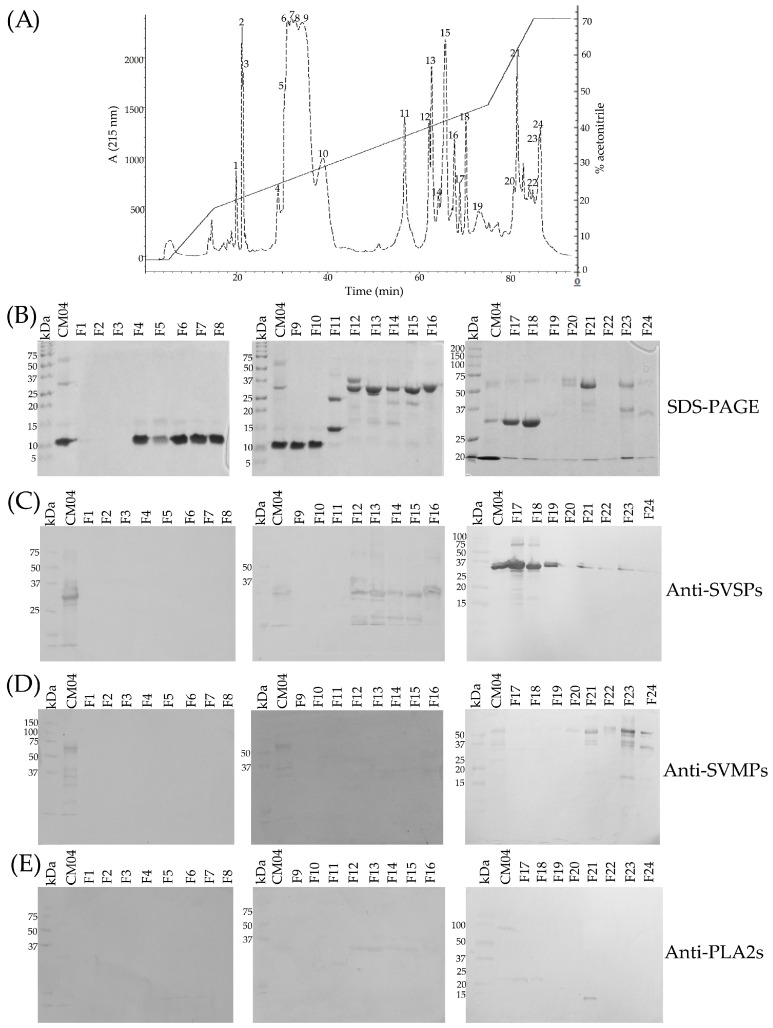
Elution profile of one representative *C. m. nigrescens* venom (CM04) by RP-HPLC, as well as SDS-PAGE and western blot analysis of its fractions. 5.5 mg of venom was fractionated on a C18 column. Twenty four fractions were collected (**A**) and separated by 15% SDS-PAGE under reducing conditions (**B**). All fractions were analyzed by western blot using polyclonal antibodies against SVSPs (**C**), SVMPs (**D**), and PLA_2_s (**E**). Antibodies were obtained from rabbit immunized with *C. simus* venom. RP-HPLC fractions 6, 7, 8, 9, and 10 were further analyzed by ESI-MS mass spectrometry to determine molecular mass of the proteins within those fractions. The acetonitrile gradient is shown in the HPLC graph and the percentage value corresponds to the right axis for each panel.

All but one venom sample was separated by RP-HPLC with the goal of visualizing differences in the protein profiles of the venoms (Figure 3 and Figure 4, Figure S1). Clear differences were detected in the RP-HPLC profiles of *C. m. nigrescens* venoms related to the TBL of snakes. In general, chromatograms tended to be less complex, in terms of total number of fractions, in venoms of larger snakes with TBL >70 cm (Figure 4, Figure S1). Sixteen venoms had peaks between 30 and 40 min of elution that were absent in the other ten venoms (Figure 3, Figure S1). The seven largest measured individuals and the two unmeasured adults lacked these peaks and the thirteen smallest snakes had them. Of the snakes between 72.5 and 80 cm, only one lacked the peaks. Because peaks eluting from 30 to 40 min were highly abundant in the venom of CM04, we used this venom for further analysis to determine the composition of the peaks observed eluting from 30 to 40 min (Figure 5A) and correspond to proteins with an apparent molecular mass close to 10 kDa from SDS-PAGE (Figure 5B). From this venom (CM04), we collected fractions that eluted from 30 to 38 min (Fractions 6, 7, 8, 9, and 10; Figure 5A) and using ESI-MS mass spectrometry obtained seven proteins with molecular masses: 5129.17 Da (from fraction 6), 5071.90 Da (from fraction 6), 5143.14 Da (from fraction 7), 5180.00 Da (from fraction 7), 4958.53 Da (from fraction 8), 5143.60 Da (from fraction 9) and 5082.50 Da (from fraction 10) (Figure S2). In addition, fractions 6–10 all induced hind limb spastic paralysis in mice, a characteristic effect generated by crotamine-like myotoxins. The i.v. LD_50_ calculated for fraction 8 was 2.23 mg/kg. Additionally, through N-terminal sequencing of fraction 8, we determined that the partial protein sequence was comprised of 34 amino acids (YKRCLKKGGHCFPKTVICLPPSSDFGKMDCRWKW) and showed 100% match to the partial sequence of crotamine 3 from *C. oreganus helleri* (Uniprot access: AEU60011.1). Thus, molecular mass, N-terminal sequence, and biological activity indicate that fractions eluting between 30 and 40 min correspond to crotamine-like myotoxins.

A second area with differences in terms of number and relative abundance of fractions among chromatograms was located between 60 to 70 min (Figure 3 and Figure 4, Figure S1). The western blot analysis of CM04 venom revealed that this area predominately contained SVSPs with a molecular mass between 25 kDa and 37 kDa (Figure 5C).

The final elution region with clear variation was the region corresponding to fractions that eluted after 78 min (Figure 3 and Figure 4, Figure S1). Using specific polyclonal antibodies against SVMPs, we determined that these fractions correspond to SVMPs with molecular masses in the range of 20 kDa to 75 kDa (Figure 5D).

**Figure 6:**
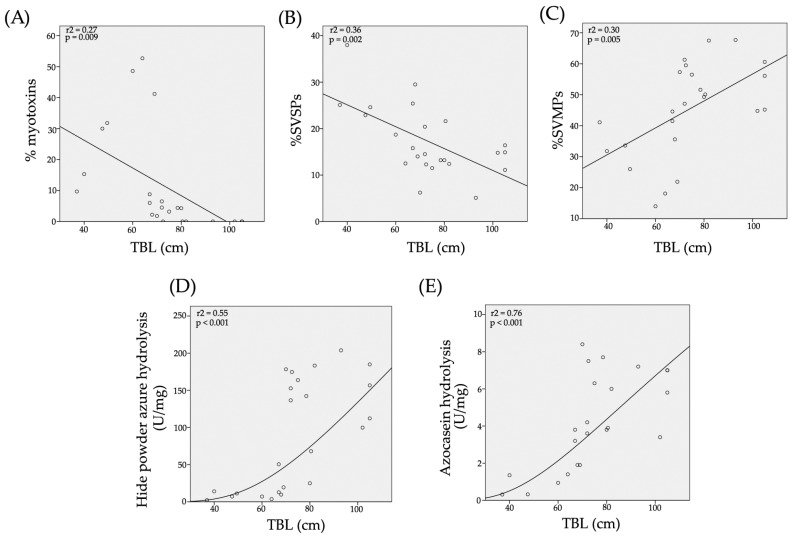
Approximate relative percentage of crotamine-like myotoxins (**A**), SVSPs (**B**), SVMPs (**C**), and proteolytic activity over hide powder azure (**D**) and azocasein (**E**) of crude venoms of *C. m. nigrescens* as a function of snake total length. Solid lines are linear curves (**A**–**C**) and sigmoidal curves (**D**,**E**).

The percentage of crotamine-like proteins in venoms, calculated from chromatograms, ranged from 1.8% to 52.7% (Table 1). Interestingly, a significant relationship (*r*^2^ = 0.27; *p* = 0.009) between the relative abundance of crotamine-like myotoxins in venoms and the TBL of snakes was identified. The percentage of myotoxins in venom became less abundant in larger snakes and completely disappears in venoms of the largest snakes (Figure 6A). On the other hand, the calculated percentage of SVSPs in *C. m. nigrescens* venoms varied from 5.1% to 38% (Table 1) and again, there was a significant relationship (*r*^2^ = 0.36; *p* = 0.002) with a reduction of SVSPs in venoms from larger snakes (Figure 6B). Finally, there was a significant relationship with TBL (*r*^2^ = 0.30; *p* = 0.005) where SVMPs increased with greater TBL (Figure 6C; Table 1).

### 2.4. Hide Powder Azure (HPA) Hydrolysis

All 27 *C. m. nigrescens* venoms analyzed had proteolytic activity on HPA. Venoms hydrolyzed the HPA in ranges from 2.2 U/mg (CM16) to 203.8 U/mg (CM13) (Table 1). A significant positive relationship (*r*^2^ = 0.55; *p* < 0.001) between snake TBL and activity on HPA was detected. As the length of snakes increased, the proteolytic activity also increased (Figure 6D). Proteolytic activity of all venoms was completely abolished with ethylenediaminetetraacetic acid (EDTA) indicating this activity is mediated by SVMPs.

### 2.5. Azocasein Hydrolysis

Similar to what was observed with the HPA substrate, the 26 venoms tested were able to hydrolyze azocasein with a significant positive relationship (*r*^2^ = 0.76; *p* < 0.001) between snake TBL and azocasein hydrolysis (Figure 6E). Venoms showed azocaseinolytic activity in the range from 0.31 U/mg (CM16) to 8.4 U/mg (CM24) (Table 1). Azocaseinolytic activity was completely inhibited with EDTA indicating that this activity is completely mediated by SVMPs.

### 2.6. Gelatin Hydrolysis

**Figure 7:**
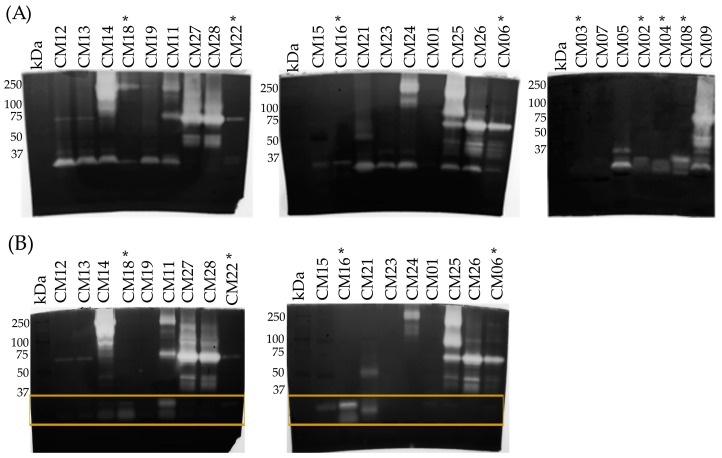
(**A**) Gelatinolytic activity of crude venoms of *C. m. nigrescens*. 1 mg/mL of gelatin was added to the 12% SDS-PAGE. 10 µg of venom were loaded per lane. Venoms of individuals with a TBL of less than 70 cm are marked with an asterisk (*). Bands containing proteolytic activity are seen as a clear band on the dark background. kDa: kilodaltons. Gels were stained with Coomassie blue. (**B**) Gelatinolytic activity of crude venoms of *C. m. nigrescens* preincubated with 20 mM EDTA for 30 min at 37 °C. Panel B shows the disappearance of activity at <37 kDa (yellow boxes), particularly for venoms CM12, CM13, CM14 (partially), CM19, CM23, CM24, CM25, CM26 and CM06. Additionally, an inhibition of the activity in the high molecular weight region (>100 kDa) was observed for the venoms CM18, CM19 and CM21. These data indicate that there are metalloproteases of type PIII and PI or PII that have been inhibited under the aforementioned conditions.

To evaluate the gelatinolytic activity of venoms, zymograms with gelatin as the substrate were carried out on 25 of the 27 venoms. Venoms degraded gelatin in different regions of the gel and with different intensity (Figure 7A). Most of the venoms hydrolyzed gelatin below 37 kDa, however, gelatinolytic activity was more heterogenous in the region above 37 kDa. For example, venoms such as CM09 and CM25 had proteolytic activity throughout the gel but many had low activity including CM01 (TBL: 105 cm), CM03 (TBL: 67 cm), CM07 (TBL: 80 cm), CM15 (TBL: 80.5 cm), CM16 (TBL: 37 cm), and CM22 (TBL: 68 cm). In general, venoms from snakes of smaller size (<70 cm) were less proteolytic than venoms of larger snakes (>70 cm); however, some venoms of individuals greater than 70 cm did not generate hydrolysis (CM01) or did in a reduced manner (CM07, CM15, and CM23) (Figure 7A). Gelatinolytic activity was only slightly inhibited with EDTA, indicating that proteases other than SVMPs (possibly SVSPs) generated most of the activity (Figure 7B).

### 2.7. Minimum Coagulant Dose (MCD-P)

Twenty-four of the twenty-seven venoms produced a fibrin clot when they were incubated with human plasma, although at different rates. Of these, three venoms (CM01, CM14, and CM24) only formed partial clots while the remaining twenty-one venoms generated complete clots (Table 1). Three venoms (CM12, CM13, and CM23) of individuals greater than 70 cm TBL lacked coagulant activity despite adding 400 µg of venom (Table 1). Although the venoms from CM19 and CM24 had procoagulant activity, it was not possible to determine their MCD-P because adding up to 400 µg of venom only produced a blood clot after 10 min of reaction. The MCD-P of venoms ranged from 10.3 µg to up to >400 µg (Table 1). Noticeably, venoms of individuals with a TBL of less than 50 cm (CM08, CM10, CM16, and CM18) showed the highest procoagulant activity with values in the range of 10.3–24.6 µg. Although, in general, venoms of smaller snakes exhibited more thrombin-like action on plasma, some venoms of snakes longer than 100 cm had coagulant activity (CM23 and CM09, MCD-P: 33.5 µg and 81.8 µg, respectively). Incubation of venoms with phenylmethylsulfonyl fluoride (PMSF) completely abolished the coagulant activity of all venoms while incubation with EDTA did not inhibit the activity. This indicates that coagulant activity is mediated by SVSPs and not by metal-dependent proteolytic enzymes like SVMPs.

### 2.8. Fibrinogenolytic Activity

**Figure 8:**
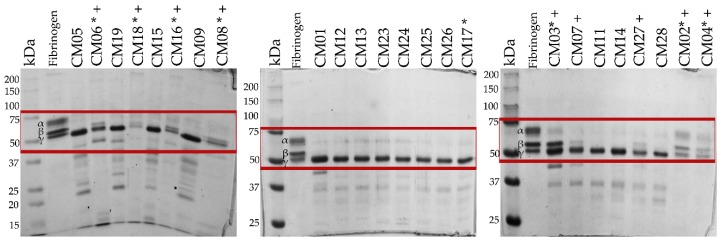
Fibrinogenolytic activity of crude venoms of *C. m. nigrescens*. 150 µg of fibrinogen were incubated with 5 µg of each venom for 15 min at 37 °C. Venoms of individuals with a TBL of less than 70 cm are marked with an asterisk (*). Venoms marked with a cross (+) generated a fibrinogen clot. Fibronogenolytic activity was visualized on 12% SDS-PAGE and it is marked with a red box. 10 µg of fibrinogen/venom mix were loaded per lane. kDa: kilodaltons. Gels were stained with Coomassie blue.

All 24 *C. m. nigrescens* venoms analyzed, hydrolyzed human fibrinogen; however, fifteen venoms hydrolyzed both the α- and β-chains of fibrinogen and nine venoms hydrolyzed only the α-chain of fibrinogen (Table 1; Figure 8). Fourteen of the fifteen venoms that cleaved both fibrinogen chains belonged to individuals with a TBL greater than 70 cm, while seven of the nine venoms that only hydrolyzed α-fibrinogen corresponded to snakes with a TBL of less than 70 cm (Table 1). Interestingly, venoms with α-chain specificity formed a fibrin clot that was not observed in venoms with α- and β-chains affinity, which indicates that thrombin-like SVSPs are responsible of the fibrinogenolytic activity in these venoms.

### 2.9. Median Lethal Dose (LD_50_)

To reduce the number of mice sacrificed, only nine representative venoms were analyzed for LD_50_. *C. m. nigrescens* venoms had toxicity in the range of 1.13 mg/kg to 4.3 mg/kg (Table 2). Five of the six venoms of snakes with a TBL of less than 70 cm were significantly more toxic (with a median LD_50_ of 1.5 mg/kg) than venoms of snakes with a TBL greater than 70 cm (with a median LD_50_ of 4.03 mg/kg; *U* = 0.00; *p* = 0.03). The one venom (CM04), corresponding to an individual with a size of 60 cm, had a LD_50_ of 4.1 mg/kg. This toxicity value was similar to those displayed by venoms of snakes with a TBL greater than 70 cm (Table 2). In addition, venoms from snakes less than 70 cm (CM03, CM04, CM06, CM08, CM16 and CM18), provoked hind limb spastic paralysis of all mice injected. This effect was not observed in the mice injected with venoms from snakes greater than 70 cm (CM09, CM15 and CM19).

### 2.10. Minimum Hemorrhagic Dose (MHD)

To reduce the number of mice sacrificed, only eight representative *C. m. nigrescens* venoms were analyzed for MHD. With the exception of the venom from CM04 which lacked hemorrhagic activity despite injecting up to 25 µg, the remaining venoms displayed hemorrhagic activity (Table 2). The most hemorrhagic venoms were CM18, CM16, and CM15 with MHD values of less than 4.0 µg, 6.1 µg and 8.3 µg, respectively. Of the remaining venoms with hemorrhagic activity, venom CM06 was the least active with a MHD of 21.9 µg (Table 2). In addition, no apparent relationship between hemorrhagic activity and TBL of snakes was observed.

### 2.11. Toxic Components in Juvenile C. m. nigrescens Venoms

**Figure 9:**
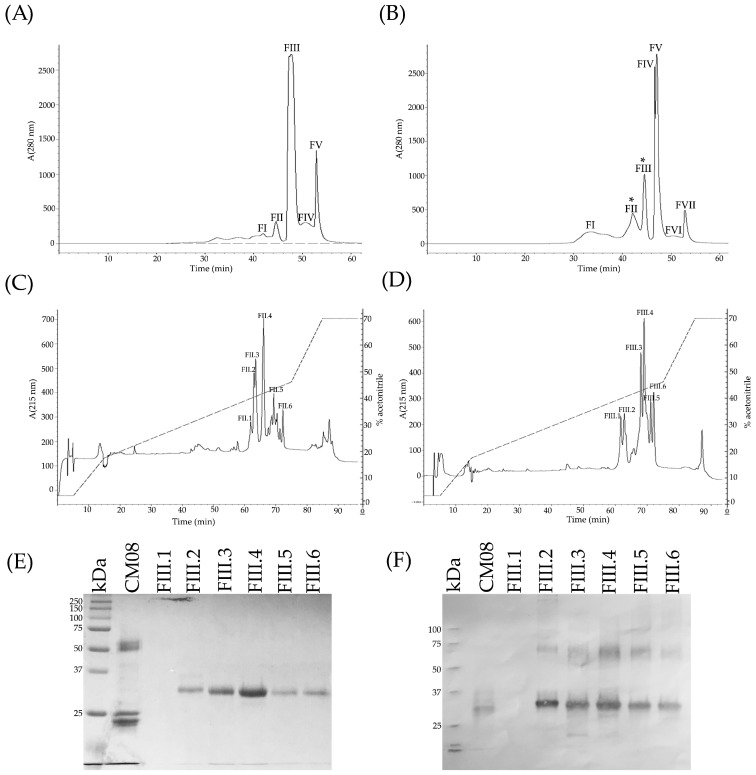
Size-exclusion chromatography of CM04 and CM08 venoms, RP-HPLC of size-exclusion fractions, and SDS-PAGE and western blot of RP-HPLC fractions. Nine and five milligrams of venoms CM04 (**A**) and CM08 (**B**), respectively, were loaded in a Cosmosil column (Diol-300, 7.5 × 600 mm) and eluted by size-exclusion chromatography. Two-hundred micrograms of FII (**C**) and FIII (**D**) fractions (the most toxic fractions to mice) from CM08 venom were further separated by RP-HPLC. RP-HPLC fractions from FIII were analyzed by SDS-PAGE (**E**) and western blot (**F**) using polyclonal antibodies against SVSPs. Two SVSPs with molecular masses in the range from 25 kD to 75 kDa were detected by westen blot, indicating that SVSPs are responsible for generating most of the toxicity in juvenile snakes.

With the goal of determining the toxic components in venoms from juvenile snakes, CM04 and CM08 venom were separated by size-exclusion chromatography. Five and seven fractions were obtained from CM04 and CM08 venoms, respectively (Figure 9A,B, respectively). Only fraction three (FIII) from CM04 venom and fractions two, three and four (FII, FIII and FIV) from CM08 venom were able to kill mice after 60 µg were injected i.v. (Table 3). However, only FIII from CM08 venom is toxic to mice after injecting only 20 µg (Table 3). Interestingly, while FIII from CM04 and FIV from CM08 caused hind limb spastic paralysis when injected in mice (indicating that they contained crotamine-like myotoxins), FII and FIII from CM08 did not induced this effect but instead induced ophisthonos and bleeding. Thus, FII and FIII from CM08 venom were further separated by RP-HPLC (Figure 9C,D, respectively). Both molecular exclusion fractions eluted proteins in the range from 60 to 72 min by RP-HPLC, however all RP-HPLC fractions lost their toxic activity in mice (possibly due to protein denaturalization by acetonitrile used in RP-HPLC) since they were unable to kill mice despite adding 25 µg. SDS-PAGE reveled that RP-HPLC fractions obtained from CM08 FIII contain a unique band between 25 kDa and 37 kDa (Figure 9E) and it was demonstrated by western blot using polyclonal antibodies against SVSPs that this band correspond to SVSPs (Figure 9F). Unexpectedly, an additional band between 50 kDa and 75 kDa was observed in the western blot, indicating that at least two SVSPs generated most of the toxicity in juvenile snakes (Figure 9F).

### 2.12. Detection of Crotoxin-Like Neurotoxins at the Protein Level

To determine if neurotoxins similar to Crotoxin/Mojave toxin were present at the protein level, an ELISA analysis was made using monoclonal antibodies against the basic subunit of crotoxin. As previously reported in literature, none of the *C. m. nigrescens* individuals were positive for neurotoxins in their venom [62,63]. As expected, the Type A *C. s. scutulatus* venom used as a control was positive for Mojave toxin.

### 2.13. Neutralization Studies

Neutralizing activity of Mexican antivenoms Antivipmyn^®^ and Faboterapico polivalente antiviperino^®^ were evaluated using venoms from four *C. m. nigrescens* individuals with different TBL (CM06, CM09, CM18 and CM19). In general, both antivenoms neutralized venoms of juvenile snakes (CM06 and CM18) better than venoms of the adults (CM09 and CM19). Antivipmyn^®^ neutralized three of the four venoms tested, with effective dose (ED_50_) values of 246.2 µL/3LD_50_, 322.1 µL/3LD_50_ and 222.0 µL/3LD_50_ for CM06, CM09 and CM18 venoms, respectively (Table 2). Similarly, Faboterapico polivalente antiviperino^®^ neutralized the same three venoms that Antivipmyn^®^ did. The ED_50_ values obtained were 158.0 µL/3LD_50_, 411.1 µL/3LD_50_ and 240.1 µL/3LD_50_ for CM06, CM09 and CM18 venoms, respectively. Neither of the two antivenoms were able to neutralize the CM19 venom despite adding up to 450 μL of each antivenom. In addition, hind limb spastic paralysis induced by the myotoxins in CM06 and CM18 venoms was not neutralized by any antivenom.

### 2.14. Immune Recognition of Antivenom

**Figure 10:**
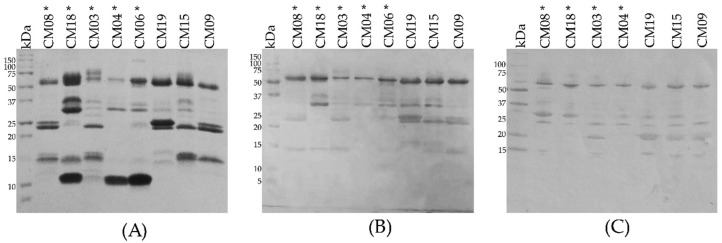
SDS PAGE and western blot analysis of representative *C. m. nigrescens* venoms. (**A**) Venoms (10 µg) were separated by 15% SDS-PAGE. Venoms (5 µg) on nitrocellulose were detected with Antivipmyn^®^ (**B**) and Faboterapico polivalente antiviperino^®^ (**C**). Venoms of individuals with a TBL of less than 70 cm are marked with an asterisk (*).

Although most proteins were recognized by the Mexican antivenoms Antivipmyn^®^ and Faboterapico polivalente antiviperino^®^ (Figure 10B,C, respectively), proteins with molecular masses lower than 20 kDa were weakly or not recognized. Importantly, crotamine-like myotoxins (~10 kDa by SDS-PAGE, Figure 10A) of juvenile venoms (CM04, CM06 and CM18) were not recognized by antivenoms, a result congruent with what was observed in the neutralization test where antivenoms did not neutralize the activity of these proteins.

## 3. Discussion

The number and relative abundance of the protein families in rattlesnake venom varies broadly at the intraspecific level. The factors commonly associated with intraspecific venom variation are the snake’s age and their geographic distribution [13,32,64,65]. In our study, we detected clear variation, in terms of relative abundance and presence/absence of proteins specifically associated with the length of individual snakes. The protein profiles of *C. m. nigrescens* venoms as demonstrated by SDS-PAGE and RP-HPLC analyses, showed marked differences in venoms from snakes of different sizes classes. The most abundant components in *C. m. nigrescens* venoms were SVMPs, SVSPs, and crotamine-like myotoxins. However, the proportion of each of these components was variable among venoms from smaller and larger snakes, where small snakes displayed more crotamine-like myotoxins and SVSPs compared to larger snakes, but the opposite occurred with SVMPs (Figure 6A–C; Table 1). In the current study, the threshold of venom change of *C. m. nigrescens* was at approximately 70 cm TBL. Snakes with a size below this threshold were, in general, more toxic and procoagulant, but less proteolytic. In addition, snakes with TBL less than 70 cm had venoms containing crotamine-like myotoxins that were reduced or absent in the snakes with a TBL greater than 70 cm. It is likely that the ontogenetic venom change observed in *C. m. nigrescens* corresponds with its sexual maturity, since this subspecies acquires sexual maturity at approximately 70 cm of TBL [51].

Crotamine is a small, non-enzymatic protein composed of about 42 amino acids (4.8 kDa) that was first isolated in the venom of *C. durissus terrificus* [66]. Among other effects, crotamine is capable of inducing spastic paralysis in the hind limbs of mice that are injected with the toxin and generates necrosis in muscle cells [67,68]. Previously, it had been reported that some rattlesnakes species, including *C. m. nigrescens*, contain crotamine-like myotoxins (e.g., α-myotoxins) in their venom [57]. In the current study, sixteen *C. m. nigrescens* venoms contained crotamine-like myotoxins in different proportion as demonstrated by RP-HPLC (Figure 3, Figure S1; Table 1). Interestingly, venoms of snakes larger than 80 cm completely lacked this small toxin (Figure 4, Figure S1; Table 1). In addition, only venoms containing crotamine-like myotoxins were able to cause hind limb spastic paralysis of all mice injected. Crotamine-like myotoxins were also visualized by SDS-PAGE as a prominent band near 10 kDa in venoms with the highest percentage of these proteins (e.g., CM02, CM04, CM06, CM08, CM16, and CM18). This apparent molecular mass obtained by SDS-PAGE is similar to those described in other analyses for this basic protein [23,47,69]. From venom CM04, we isolated at least seven crotamine-like myotoxin isoforms with molecular masses ranging from 4958.53 Da to 5180.00 Da and obtained the N-terminal sequence of one of them. Presence of multiple forms of crotamine-like myotoxins in the same venom has been reported in other *Crotalus* venoms and these isoforms can differ only in few amino acids [70], what could explain the variation in the molecular masses of the isoforms detected in *C. m. nigrescens* venom. Additionally, the i.v. LD_50_ calculated for one of these isoforms was 2.23 mg/kg, a value within the range reported for the toxicity of crotamine (from 0.07 to 32.76 mg/kg) [67]. Presence of myotoxins in the venom of juvenile snakes and their consequent reduction in adult snakes has been reported in *C. adamanteus* and *C. tzabcan* (the juvenile has 34% while the adult has 18.5% of myotoxins) [15,49]. However, an inverse pattern has been detected in *C. oreganus concolor* and *C. viridis viridis*, where venoms of adult individuals expressed a greater amount of myotoxins compared to juvenile snakes [14,71].

Thrombin-like SVSPs (TL-SVSPs) are able to hydrolyze both α and β chains of fibrinogen, releasing fibrinopeptides A and B, respectively, but most of them preferentially act upon only on one of the fibrinogen chains [72]. On the other hand, fibrinogenases can be classified as α-fibrinogenases and β-fibrinogenases, where the first group hydrolyses preferentially (although not exclusively) α-chain of fibrinogen, while the second group has more affinity for β-chain of fibrinogen. Unlike TL-SVSP, fibrinogenases do not release fibrinopeptides A or B, do not induce fibrin clot formation and most of them are SVMPs [73,74]. All venoms of *C. m. nigrescens* analyzed had fibrinogenolytic activity. Interestingly, while fifteen venoms (most of them corresponding to snakes with a TBL greater than 70 cm) hydrolyzed both the α- and β-chains of fibrinogen, ten venoms (most of them belonging to snakes with a TBL of less than 70 cm) hydrolyzed only the α-chain of this plasmatic protein and generated a fibrin clot. In addition, *C. m. nigrescens* venoms of snakes with a TBL less than 60 cm were the most procoagulant on human plasma with MCD-P values in the range of 10.3 µg to 24.6 µg. Pre-incubation of venoms with SVSPs inhibitor (PMSF) completely inhibited the coagulant activity of venoms, indicating that this activity is mediated solely by SVSPs. Similar results has been reported in species such as *C. durissus cumanensis* [18] and *C. d. terrificus* [75] where venoms from neonate or juvenile snakes had MCD-P lower (more coagulant) than venoms of adult snakes. These results suggest that fibrinogenases are responsible for activity in *C. m. nigrescens* venoms breaking both α- and β-chains of fibrinogen, while TL-SVSPs would be responsible of the activity in venoms with affinity only for the α-chain of fibrinogen.

Within *C. m. nigrescens*, there was also an increase in the relative abundance of SVMPs in venom as the snakes increase in TBL. Similarly, a positive significant relationship was found between TBL of snakes and the proteolytic activity on HPA and azocasein of their venoms. Specifically, the longer the snake, the greater the proteolytic activity. Thus, taking into account that EDTA completely inhibited the proteolytic activity over both substrates, it is likely that the increase in proteolytic activity in venoms as the snakes get longer is related to the increase in the content of SVMPs in venom. Interestingly, gelatinolytic activity did not increase with the length of snakes and was not completely inhibited by EDTA which indicates that not only metal dependent proteolytic enzymes are associated with this activity. An increase of SVMPs abundance and proteolytic activity with increasing snake size has been reported in venom of other rattlesnakes such as *C. oreganus helleri*, *C. o. oreganus*, *C. polystictus*, and *C*. *simus* [19,21,23]. Though, species such as *C. o. concolor* and *C. viridis viridis* exhibit a reduction in the amount and activity of SVMPs as the snakes increase their longitude [14,71].

It has been proposed that juvenile snakes require toxic venoms to ensure rapid prey immobilization when prey abundance is limited and since eating frequently allows them to reach the adult stage (a less vulnerable stage against predators) more rapidly [15,76]. Comparatively, for adults that feed on larger prey, having venoms rich in proteases (e.g., metalloproteases) is more important to properly digest their prey than having toxic venoms [15,76]. In *C. m. nigrescens*, most of the venoms of snakes with a TBL of less than 70 cm were more toxic and were equally hemorrhagic compared to venoms of snakes with larger size. The main components related to toxicity in small *C. m. nigrescens* snakes were SVSPs (with molecular masses between 25 kDa and 75 kDa) and coincidentally, venom of juvenile snakes contained, in general, more SVSPs than venom of adult snakes. In addition, mice injected with these SVSPs showed opisthotonos and bleeding. A group of TL-SVSPs (e.g., gyroxin, crotalase, ancrod, leucurobin, and LM-TL) with molecular masses in the range from 30 kDa to 60 kDa produce temporary episodes characterized by opisthotonos and rotations around the long axis of mice [77,78] and can have high toxicity (e.g., LD_50_ of 0.07 µg/g in mice) [77]. Our results suggest that a combination of myotoxic (crotamine-like myotoxins), procoagulant (TL-SVSPs) and hemorraghic (SVMPs) components could induce faster paralysis and death in prey of juveniles *C. m. nigrescens*, compared to venoms of adult snakes that lack myotoxins and have limited or no procoagulant activity.

Although venom variation related to age of snakes was demonstrated for *C. m. nigrescens*, venom variation related to geographic distribution was not as clear. In general, *C. m. nigrescens* individuals with similar TBL but from different localities from Mexico displayed similar venoms in terms of protein profile, toxicity, and proteolytic activity related to SVMPs. Gelatinolytic, fibrinogenolytic, and coagulant activities were more variable among venoms but even in these activities no patterns were associated with geographic distribution. Rattlesnake venoms can be classified in two groups according to their toxicity and SVMP mediated proteolytic activity, where Type I corresponds to venoms with low or moderate toxicity and high proteolytic activity, while Type II venoms are more toxic and less proteolytic [76]. Nevertheless, this classification can be made more complex if we take into account that the main components of these venom groups, such as neurotoxins, SVMPs, and myotoxins, can vary widely in terms of presence and relative abundance [25,34]. Further, some rattlesnake species, such as *C. helleri*, *C. horridus*, *C. scutulatus*, and *C. simus,* can have populations with Type I, Type II, and/or Type I + II venoms [31,33,34,47,79,80]. Taking into account their low toxicity (compared to neurotoxic venoms), high proteolytic activity and lack of the Mojave toxin (in agreement with other reports [62,63]), *C. m. nigrescens* venoms would be classified exclusively as Type I.

Both Mexican antivenoms (Antivipmyn^®^ and Faboterapico polivalente antiviperino^®^) effectively neutralized lethal activity of two juvenile *C. m. nigrescens* venoms. However, the western blot analysis revealed that both Mexican antivenoms did not recognize proteins with molecular mass close to 10 kDa (e.g., crotamine-like myotoxins) from juvenile *C. m. nigrescens* venoms, suggesting that both antivenoms lack specific antibodies against these small proteins. Another possibility is that epitopes in small proteins (~10 kDa) were disrupted due to the reduced conditions used in SDS-PAGE, so that antivenoms were not able to recognize them. However, neither antivenom neutralized the activity of crotamine-like myotoxins in vivo, suggesting that antivenoms did not recognize and were unable to neutralize denatured or native small proteins. In contrast, both antivenoms had difficulties neutralizing lethality of venoms of adult snakes; one of the adult venoms was not neutralized by either antivenom. It has been reported that venoms that have high LD_50_s need greater volume of antivenom to neutralize the venom which could explain the lack of neutralization for one adult. For example, *C. culminatus* venom with LD_50_s ranging from 66 to 162 µg/mouse is not neutralized with volumes less than equal to 460 µL of antivenom [81]. The opposite relationship has been reported for snake species such as *Bothrops asper* and *C. durissus durissus*, where antivenoms from Costa Rica were less effective neutralizing newborn snake venoms compared to adult ones [17,82]. Other studies have demonstrated that some experimental antivenoms from Costa Rica reacted weakly with proteins with molecular masses lower than 20 kDa of *B. asper* venom [83]. Nevertheless, CroFab^®^ antivenom did recognize myotoxin a of juvenile and adult *Crotalus viridis viridis* venoms [71], indicating that a representative selection of immunization mixtures in antivenoms production is crucial to have antibodies able to neutralize intraspecific venom variation. Results suggest that Mexican antivenoms could have problems neutralizing human envenomation by some adult *C. m. nigrescens* and a higher than normal dose would be necessary to effectively neutralize the venom.

## 4. Conclusions

It has been demonstrated that rattlesnakes species such as *C. o. helleri*, *C. o. oreganus*, *C. o. concolor,* and *C. v. viridis* undergo ontogenetic changes in their diet, in which they go from feeding mainly on ectothermic animals (e.g., lizards) in juvenile stage to consume endothermic animals (e.g., mammals) in the adult stage [14,19,71]. These same species undergo a marked change in the composition of their venom in relation to age, indicating that diet plays in important role in the regulation of expression of protein families in venom. Based on what is known in the *C. molossus* complex about diet [54,55], it is likely that venom compositional changes are due to diet differences through time.

Therefore, it is possible to hypothesize that the ontogenetic venom variation observed in the current study could be associated, at least in part, with differences in prey consumed by this subspecies during its life cycle. However, it is necessary to corroborate this hypothesis with a diet analysis.

Based on this study and previous studies on rattlesnake venom variation, rattlesnakes appear to evolve unique strategies given different environmental pressures exerted on them. In the case of *C. m. nigrescens*, an ontogenetic venom change seems to be more advantageous than changes in the venoms of adult individuals across their distribution. It is likely that further analysis of venom in the remaining subspecies (*C. m. estebanensis*, *C. m. molossus,* and *C. m. oaxacus*) and species (*C. ornatus*, *C. basiliscus*, and *C. totonacus*) in the *Crotalus molossus* species complex will find the same pattern that we did in *C. m. nigrescens*.

## 5. Materials and Methods

### 5.1. Ethics Statement

We followed the guidelines described by the live animal use committees of the Facultad de Ciencias Biológicas at UJED, the Universidad Nacional Autónoma de México, and the Secretaría de Medio Ambiente y Recursos Naturales (SEMARNAT) of Mexico as well as developing our protocols in accordance with the American Society of Ichthyologists and Herpetologists guidelines for use of live amphibians and reptiles. SEMARNAT issued collecting permits (SGPA/DGVS/002288/18, 01090/17 and 03562/15; 13 March 2018; 10 February 2017; 7 April 2015, respectively, for samples collected in Mexico.

### 5.2. Crotalus Molossus Nigrescens Sampling

Snakes were captured in the field, venom was sampled, and then the animal was immediately released after venom extraction and measurements were taken. Samples were obtained from twenty-seven *C. m. nigrescens* from eight states (Aguascalientes, Coahuila, Durango, Jalisco, Nuevo León, San Luis Potosí, Tamaulipas and Zacatecas) in Mexico (Table 1). When possible, total body length (TBL) was measured and sex determined for each snake. Venom was extracted manually by allowing each snake to bite sterile 100 mL plastic containers covered with parafilm. Venoms were centrifuged at 14,000 rpm for 1 min to remove debris and the supernatant was lyophilized and stored at −20 °C until use. Tests using mice were carried out only with representative venoms to reduce the number of animals sacrificed.

### 5.3. Protein Concentration Determination

The protein concentration of each venom was obtained using the Pierce^®^ Bicinchoninic Acid (BCA) Protein Assay (Thermo Scientific, Rockford, IL, USA) following the manufacturer´s instructions and using bovine serum albumin (BSA) as a standard.

### 5.4. SDS-PAGE

15% polyacrylamide gels were made on a Miniprotean III system (Bio-Rad, Hercules, CA, USA) using the discontinuous system. Fifteen micrograms of each venom were dissolved in sample buffer (50 mM Tris-HCl, pH 6.8, 25% SDS, 10% glycerol, and 0.002% bromo-phenol blue) and then we added 5% *β*-mercaptoethanol. Each of the 27 samples were boiled for 5 min and run at 120 V. Gels were stained with 0.2% Coomassie brilliant blue R-250, 10% acetic acid, and 25% methanol for 8 h and rinsed in 10% acetic acid and 10% methanol [33]. Standard molecular mass markers (Bio-Rad, Carlsbad, CA, USA) were used as references.

### 5.5. Reverse-Phased High Performance Liquid Chromatography HPLC

Separation of 26 venom samples by RP-HPLC was carried out on an analytic C18 reverse-phase column (Vydac^®^, Deerfield, IL, USA, 218 TP 4.6 mm × 250 mm) using an Agilent 1100 chromatograph. One milligram of each venom was dissolved in 1.7 mL of water containing 0.1% trifluoroacetic acid (TFA). Elution was performed as described by Castro et al. [47] at 1 mL/min by applying a gradient toward solution B (acetonitrile, containing 0.1% TFA), as follows: 0% B for 5 min, 0 to 15% B over 10 min, 15 to 45% B over 60 min, 45 to 70% B over 10 min, and 70% B for 9 min. Proteins were detected at 215 nm.

### 5.6. Identification of Protein Families in Representative C. m. nigrescens Venom by Western Blot

In order to determine the identity of the main fractions obtained from the RP-HPLC, fractions and whole CM04 venom were analyzed by western blot using polyclonal antibodies against SVMPs, SVSPs and PLA_2_s. Antibodies against SVMPs, SVSPs, or PLA_2_s were obtained from serum of rabbits hyperimmunized with *C. simus* venom. An immunopurification step was carried out to get specific antibodies against every protein family. Depending of the antibodies, a final step of negative immunopurification was used to remove antibodies with cross-reactivity or residual cross-contamination to SVMPs, SVSPs, or PLA_2_s.

Western blot was carried out as follow. Two micrograms of each fraction and five micrograms of whole venom were separated by 15% SDS-PAGE. After electrophoresis, proteins were transferred to a nitrocellulose membrane (Trans-blot 0.45 µm, Bio-Rad, Merck, Cork, Ireland) using a model HEP-1^®^ semi-dry immunotransference chamber (Thermo Scientific, Carlsbad, CA, USA). After transference at 420 mA for 1 h, the membrane was blocked with 5% non-fat dry milk diluted in TBST buffer (10 mM Tris-HCl, pH 7.5, 150 mM NaCl, and 0.05% Tween-20, pH 8.4) for 2 h. The membrane was then rinsed three times with TBST and incubated by shaking gently for 1 h at room temperature with antibodies against SVMPs, SVSPs, or PLA_2_s diluted 1 µg/mL in TBST. After three washes with TBST, the membrane was incubated at room temperature for 1 h with goat antibodies against rabbit IgG conjugated to alkaline phosphatase (Invitrogen, Eugene, OR, USA) diluted 1:7000. The membrane was again rinsed with TBST and then developed by adding the BCIP/NPT substrate for alkaline phosphatase (Invitrogen, Camarillo, CA, USA) [33].

### 5.7. Molecular Mass and N-Terminal Sequence Determination of Crotamine-Like Myotoxins

We determined the intact masses of crotamine-like myotoxins of venom from one individual (CM04) using ESI-MS on an LCQ Fleet Ion Trap Mass Spectrometer. Amino-terminal sequencing of one crotamine-like myotoxin (RP-HPLC fraction 8) was determined by automated Edman degradation on a PPSQ-31A Protein Sequencer (Shimadzu, Tokyo, Japan).

### 5.8. Hide Powder Azure (HPA) Hydrolysis

We used the Hide Powder Azure (HPA) hydrolysis assay to determine proteolytic activity for each of the 27 samples. Hydrolysis of HPA was determined by adding 25 µg of venom to 1 mL of a solution containing 5 mg of HPA in 0.1 M Tris-HCl, pH 8.0. After 2 h of incubation, the reaction was stopped by centrifugation at 14,000 rpm for 5 min. The supernatant was obtained and the absorbance at 595 nm was measured. The absorbances of samples corresponding to HPA solutions incubated with 0.1 M Tris-HCI, pH 8.0, were subtracted from the absorbances of venom-containing samples. Analyses were conducted in triplicate. A standard curve of HPA hydrolysis was created by adding 50 µL of trypsin (2 mg/mL) to three concentrations of HPA (2, 4 and 6 mg/mL) and venom enzymatic activity was calculated using the standard curve. A unit of enzymatic activity (U) was defined as the amount of venom necessary to digest 1 mg of HPA in a 2 h period at room temperature and reported as specific activity (U/mg) [33].

### 5.9. Azocasein Hydrolysis

Proteolytic activity of 26 venoms was evaluated using azocasein (Sigma-Aldrich, St. Louis, MO, USA) as the substrate, as described by Castro et al. [47]. Ten micrograms of venom, dissolved in 20 μL 0.15 M NaCl, were added to 100 μL of 10 mg/mL azocasein, dissolved in 50 mM Tris0.15 M NaCl, 5 mM CaCl_2_, pH 8.0. After 60 min of incubation at 37 °C, the reaction was stopped by adding 200 μL of 5% trichloroacetic acid. The mixture was centrifuged and 150 μL of the supernatant was added to 150 μL of 0.5 M NaOH. Absorbances were recorded at 450 nm. The absorbances of samples corresponding to azocasein solutions incubated with 0.15 M NaCl were subtracted from the absorbances of venom-containing samples. One unit of proteolytic activity was defined as a change of 0.2 in absorbance per min.

### 5.10. Gelatinolytic Activity

Gelatin zymography was performed in 12% SDS-PAGE gels containing 1 mg/mL gelatin (Bio-Rad, Carlsbad, CA, USA) for all 25 samples. The venoms (10 µg) were solubilized (5:1 venom/buffer) in sample buffer (50 mM Tris-HCl, pH 6.8, 25% SDS, 10% glycerol, and 0.002% bromophenol blue) in the absence of β-mercaptoethanol and without boiling. After electrophoresis at 120 V, the gels were placed sequentially in renaturing buffer (5% Triton X-100 in 0.1 M Tris-HCl, pH 8) for 2 h and developing buffer (0.1 M Tris-HCl, pH 8) for 10 min. Gels were then incubated in developing buffer at 37 °C for 12 h with gentle agitation. Gels were stained with staining solution (0.2% Coomassie brilliant blue R-250, 10% acetic acid, and 25% methanol) and destained with 10% acetic acid plus 10% methanol. Standard molecular mass markers (Bio-Rad, Carlsbad, CA, USA) were used as references [46,48].

### 5.11. Minimum Coagulant Dose Plasma (MCD-P)

For all samples, various amounts of venom (from 10 µg to 400 µg), dissolved in 100 μL 0.15 M NaCl, was added to 200 μL of citrated human plasma, previously incubated at 37 °C. Clotting timeswere recorded and coagulant activity was expressed as the Minimum Coagulant Dose in plasma (MCD-P) defined as the dose of venom inducing clotting in 60 s [84]. As a negative control, 100 µL of 0.15 M NaCl without venom was added to citrated human plasma.

### 5.12. Fibrinogenolytic Activity

Fibrinogenolytic activity of the venoms was tested using the procedure described by Borja et al. [46]. Human fibrinogen (150 µg; Sigma, St. Louis, MO, USA) was incubated with 24 individual venoms (5 µg) in 0.05 M Tris-HCl buffer, pH 8.0, at 37 °C for 15 min. The reaction was stopped by adding reducing SDS-PAGE sample buffer (50 mM Tris-HCl, pH 6.8, 25% SDS, 10% glycerol, 0.002% bromophenol blue, and 5% β-mercaptoethanol) and heating to 100 °C for 5 min. Fibrinogenolytic activity was demonstrated by 12% SDS-PAGE. As a negative control, human fibrinogen without venom was added to gels. Standard molecular mass markers (Bio-Rad, Carlsbad, CA, USA) were used as references.

### 5.13. Enzymatic Inhibition Analysis

To evaluate the influence of SVMPs in proteolytic activities over HPA, azocasein, gelatin, and human plasma, venoms were preincubated with 20 mM EDTA for 30 min at 37 °C before carrying out the analyses [40]. Similarly, coagulant activity over human plasma was carried out after preincubating venoms with 5 mM PMSF (inhibitor of serine proteases) for 15 min at 37 °C [85]. A concentrated stock of PSMF (167 mM) was prepared in 100% isopropanol. Then 3 µL of inhibitor (167 mM) were added to 97 µL of PBS containing venom samples and incubated for 10 min before being added to human plasma. A control of 3 µL of 100% isopropanol without inhibitor was used to demonstrate that isopropanol itself does not inhibit enzyme activity.

### 5.14. Median Lethal Dose (LD_50_)

Nine representative venoms were dissolved in phosphate buffer solution (PBS), pH 7.2. The lethal dose was determined by injecting different quantities of venom (from 10 µg to 150 µg) diluted in a total volume of 0.5 mL into the caudal vein of male and female ICR-CD1 mice (18 to 20 g) in groups of three [86]. The percentage of dead mice 24 h after inoculation was plotted against the logarithm of the quantity of venom injected and analyzed with nonparametric methods using the program GraphPad Prism V4 (GraphPad Sofware, La Jolla, CA, USA, 2005) to determine the LD_50_ value for each venom.

### 5.15. Minimum Hemorrhagic Dose (MHD)

Different quantities of eight representative venoms (from 5 µg to 25 µg) were injected intradermally, in a volume of 50 µL PBS, to groups of three (male and female) ICR-CD1 mice (25–28 g); 3 h later, they were sacrificed by CO_2_, their skin removed, and the area of the hemorrhagic spot was measured. The minimum hemorrhagic dose was defined as the dose of venom which induced a lesion of 10 mm diameter [87].

### 5.16. Detection of Crotoxin-Like Neurotoxins at the Protein Level

Detection of crotoxin-like neurotoxins was carried out using the procedure previously described to detect neurotoxins in *C. s. scutulatus* venoms [33]. Venom from two Mojave Rattlesnakes with Type A and Type B venom from Arizona were used as control.

### 5.17. Neutralization Studies

Neutralization studies were carried out using a modification of the procedure described by Gutierrez et al. [88]. Venoms of four *C. m. nigrescens* individuals with different TBL (CM06, CM09, CM18 and CM19) were challenged against two Mexican antivenoms: Antivipmyn^®^ manufactured by Bioclon (Lot. B-9F-22) and Faboterapico polivalente antiviperino^®^ manufactured by Birmex (Lot. FV030A). Amounts equivalent to 3 times the LD_50_ of each venom were incubated with different volumes of each antivenom (from 50 µL to 450 µL of antivenom) dissolved in PBS in a constant final volume of 500 µL for 30 min at 37 °C. Next, 500 µL of solution containing venom/antivenom were i.v. injected in groups of three (male and female) ICR-CD1 mice (18 to 20 g) and the survival percentage 24 h after inoculation was recorded. As controls, groups of three mice were injected with venom alone (positive) or antivenom alone (negative). Neutralization was expressed as Effective Dose 50% (ED_50_), the volume of antivenom that prevents the death of half of the injected population [81].

### 5.18. Immune Recognition of Antivenom

To observe the immune recognition of both Mexican antivenoms (Antivipmyn^®^ and Faboterapico polivalente antiviperino^®^) towards the components of eight representative *C. m. nigrescens* venoms (CM03, CM04, CM06, CM08, CM09. CM15, CM18, CM19), 10 µg of whole venom was separated by 15% SDS-PAGE. After electrophoresis, proteins were transferred to a nitrocellulose membrane (Trans-blot 0.45 µm, Bio-Rad, Merck, Cork, Ireland) using a model HEP-1^®^ semi-dry immunotransference chamber (Thermo Scientific). After transference at 420 mA for 1 h, the membrane was blocked with 5% non-fat dry milk diluted in TBST buffer (10 mM Tris-HCl, pH 7.5, 150 mM NaCl, and 0.05% Tween-20, pH 8.4) for 2 h. The membrane was then rinsed three times with TBST and incubated by shaking rently for 1 h at room temperature with Antivipmyn^®^ or Favoterapico polivalente antiviperino^®^ diluted 1:800 in TBST. After three washes with TBST, the membrane was incubated at room temperature for 1 h with goat antibodies against house IgG conjugated to alkaline phosphatase (Jackson Immuno Reserach) diluted 1:7000. The membrane was again rinsed with TBSTand then developed by adding the BCIP/NPT substrate for alkaline phosphatase (Sigma^®^).

### 5.19. Statistical Analysis

The SigmaPlot^®^11 (Systat Software Inc. (SSI), San Jose, CA, USA, 2008) statistical program was used to determine the regression that best fit our data. We regressed the relative abundance of myotoxins, SVSP, and SVMPs in the venom to the total body length of the snake and evaluated the fit with linear curves. The relationship of proteolytic activity of venoms to snake total body length was assessed by sigmoidal curves. Differences in LD_50_ between snakes with a TBL less than and greater than 70 cm were compared by using the Mann-Whitney *U* test.

## Figures and Tables

**Table 1:** Biologic and biochemical properties of *C. m. nigrescens* venoms from Mexico sorted by total body length (TBL) from smallest to largest.

ID	Sex	TBL (cm)	Locality	% of Crotamine-Like Myotoxins in Venom	% of SVMPs in Venom	% of SVSPs in Venom	HPA Hydrolysis (U/mg)	Azocasein Hydrolysis (U/mg)	MCD-P (µg of Venom)	Fibrinogenolytic Activity
CM16	F	37	La Ascensión, N. L.	9.7	41.1	25.1	2.2 ± 0.04	0.31 ± 0.02	13.5	α
CM08	M	40	Ignacio López Rayón, Dgo.	15.3	31.8	38.0	14.0 ± 0.3	1.35 ± 0.01	10.3	α
CM18	F	47.5	Genaro Codina, Zac.	30.0	33.6	22.9	7.0 ± 0.3	0.32 ± 0.01	14.7	α
CM10	ND	49.5	Real de Catorce, S. L. P.	31.8	26.0	24.6	11.1 ± 0.1	ND	24.5	ND
CM04	F	60	Mapimí, Dgo.	48.6	14.0	18.7	7.0 ± 0.7	0.94 ± 0.08	416	α
CM02	M	64	Pedriceña, Dgo.	52.7	18.1	12.5	3.6 ± 0.08	1.4 ± 0.06	36	α
CM03	F	67	Matamoros, Coah.	8.8	44.6	15.8	12.8 ± 0.6	3.8 ± 0.13	390	α
CM17	M	67	Guadalupe del Carnicero, S. L. P.	6.0	41.5	25.4	50.5 ± 2.9	3.2 ± 0.01	301	α and β
CM22	F	68	Miquihuana, Tam.	2.2	35.6	29.5	9.6 ± 0.08	1.9 ± 0.06	44	ND
CM06	F	69	Agua Puerca, Dgo.	41.2	21.9	14.0	19.4 ± 0.7	1.9 ± 0.03	151	α
CM24	M	70	Matamoros, Coah.	1.8	57.32	6.22	178.3 ± 4.0	8.4 ± 0.27	>400 *	α and β
CM11	M	72	Tepezalá, Ags.	6.5	47.1	20.4	136.5 ± 4.2	4.2 ± 0.18	43.3	α and β
CM14	M	72	Genaro García, Zac.	4.5	61.3	14.5	152.7 ± 2.5	3.6 ± 0.19	271 *	α and β
CM05	M	72.5	Nazas, Dgo.	0	59.5	12.3	174.7 ± 2.6	7.5 ± 0.27	75	α and β
CM19	F	75	Genaro Codina, Zac.	3.2	56.5	11.5	163.8 ± 11.0	6.3 ± 0.16	>400	α and β
CM25	F	78.5	Agua Puerca, Dgo.	4.4	51.6	13.2	142.3 ± 4.3	7.7 ± 0.25	182	α and β
CM07	M	80	Barreal de Guadalupe, Coah.	4.3	49.3	13.2	24.9 ± 1.8	3.8 ± 0.15	350	α
CM15	M	80.5	La Ascensión, N. L.	0	50.1	21.6	68.0 ± 1.33	3.9 ± 0.01	470	α and β
CM12	M	82	Genaro García, Zac.	0	67.5	12.4	183.3 ± 6.5	6.0 ± 0.05	0 ^†^	α and β
CM13	M	93	Genaro García, Zac.	0	67.7	5.1	203.8 ± 3.2	7.2 ± 0.21	0 ^†^	α and β
CM27	F	102	Milpillas de Arriba, Ags.	0	44.8	14.8	99.8 ± 7.4	3.4 ± 0.20	33.5	α
CM01	M	105	Jimulco, Coah.	0	56.1	16.4	112.2 ± 4.1	5.8 ± 0.14	269*	α and β
CM09	M	105	Emiliano Zapata, Dgo.	0	45.2	14.9	184.9 ± 6.3	7.0 ± 0.12	81.8	α and β
CM23	M	105	Matamoros, Coah.	0	60.6	11.1	156.7 ± 12.0	7.0 ± 0.21	0 ^†^	α and β
CM21	M	ND	Ojuelos, Jal.	0	61.8	14.5	180.3 ± 15.5	4.4 ± 0.10	177	ND
CM26	M	ND	La Loma, Dgo.	ND	ND	ND	134.6 ± 5.6	6.6 ± 0.40	267	α and β
CM28	ND	ND	Sierra del Laurel, Ags.	0	54.3	16.7	50.6 ± 4.4	3.8 ± 0.06	45.3	α and β

ND: not determined due to lack of venom; TBL: total body length; Sex: F (female); M (male); MCD-P: Minimum coagulant dose plasma; HPA: hide powder azure; SVMP: snake venom metalloproteinases; Ags. Aguascalientes; Coah: Coahuila; Dgo: Durango; Jal: Jalisco; N. L.: Nuevo León; S.L.P.: San Luis Potosí; Tam: Tamaulipas; Zac: Zacatecas; ^†^ No coagulant activity was observed even after adding up to 400 µg of venom; * Only partial clot was detected.

**Table 2:** Toxicity and minimum hemorrhagic dose (MHD) of representative *C. m. nigrescens* venoms from Mexico.

ID	TBL (cm)	LD_50_ (mg/kg)	MHD (µg)	Neutralization (ED_50_) Antivipmyn^®^ (µLAV/3LD_50_)	Neutralization (ED_50_) Faboterapico Polivalente Antiviperino^®^ (µLAV/3LD_50_)
CM16	37	1.26 *	6.10	ND	ND
CM08	40	1.71 *	19.30	ND	ND
CM18	47.5	1.13 *	4.0	222.0 (204.4–241.1) **	240.1 (184.1–313.1) **
CM04	60	4.10 *	>25	ND	ND
CM03	67	1.71 *	ND	ND	ND
CM06	69	1.72 *	21.90	246.2 (214.1–283.0) **	158.0 (121.7–205.1) **
CM19	75	4.20	10.26	>450	>450
CM15	80.5	3.60	8.30	ND	ND
CM09	105	4.30	10.95	322.1 (309.3–335.3)	411.1 (401.7–420.7)

* Provoked hind limb spastic paralysis of all mice injected during LD_50_ tests. ** Mice presented hind limb spastic paralysis during neutralization studies. ED_50_: effective dose 50%: neutralizing potency expressed as microliters of antivenom that neutralized 3 times the median lethal dose (LD_50_). >450: Mice did not survive with volumes less than or equal to 450 µL. ND: not determined.

**Table 3:** Toxicity on mice of size-exclusion chromatography fractions from CM04 and CM08 venoms.

Fraction/Venom	Mortality with Different Amounts of Venom (µg/mice)					
	CM04 (60)	CM08 (60)	CM04 (45)	CM08 (45)	CM04 (20)	CM08 (20)
FI	0/3	0/3	NA	NA	NA	NA
FII	0/3	3/3	NA	1/3	NA	0/3
FIII	3/3	3/3	1/3	2/3	0/3	1/3
FIV	0/3	3/3	NA	1/3	NA	0/3
FV	0/3	0/3	NA	NA	NA	NA
FVI	NA	0/3	NA	NA	NA	NA
FVII	NA	0/3	NA	NA	NA	NA

NA: Not applicable.

## Supplementary Materials

The following are available online at http://www.mdpi.com/2072-6651/10/12/501/s1, Figure S1. Reverse-phase HPLC chromatograms of remaining *C. m. nigrescens* venoms of snakes with different total body length (TBL). An analytic C18 reverse-phase column (Vydac^®^, Deerfield, IL, USA, 218 TP 4.6 mm × 250 mm) was used. Retention time is along the x axis for each panel and labeled every twenty min. Proteins were detected at 215 nm and absorbance is indicated on the left axis. The acetonitrile gradient is shown in the HPLC graph and the percentage value corresponds to the right axis for each panel. The regions where crotamine-like myotoxins (Myo), snake venom serine proteases (SVSPs), and snake venom metalloproteinases (SVMPs) elute are illustrated in blue, green and red, respectively. Figure S2. Mass spectrometry of crotamine-like myotoxins. RP-HPLC fractions 6 (A), 7 (B), 8 (C), 9 (D) and 10 (E) obtained from CM04 venom were analyzed using ESI-MS on an LCQ Fleet Ion Trap Mass Spectrometer. Molecular masses calculated were 5129 Da (A), 5071 Da (A), 5143 Da (B), 5180 Da (B), 4958 Da (C), 5143 Da (D), and 5082 Da (E).
