# Supplementary material for: Ontogenetic Change in the Venom of Mexican Black-Tailed Rattlesnakes (Crotalus molossus nigrescens)
Source: Toxins (Basel). 2018 Dec 1;10(12):501. doi: 10.3390/toxins10120501 (PMC6315878; doi:10.3390/toxins10120501)
Supplement: Supplementary file 1 [file toxins-10-00501-s001.pdf]

Supplementary Materials: Ontogenetic Change in the Venom of Mexican Black-Tailed Rattlesnakes (*Crotalus molossus nigrescens*)

Miguel Borja, Edgar Neri-Castro, Rebeca Pérez-Morales, Jason L. Strickland, Roberto Ponce-López, Christopher L. Parkinson, Jorge Espinosa-Fematt, Jorge Sáenz-Mata, Esau Flores-Martínez, Alejandro Alagón and Gamaliel Castañeda-Gaytán

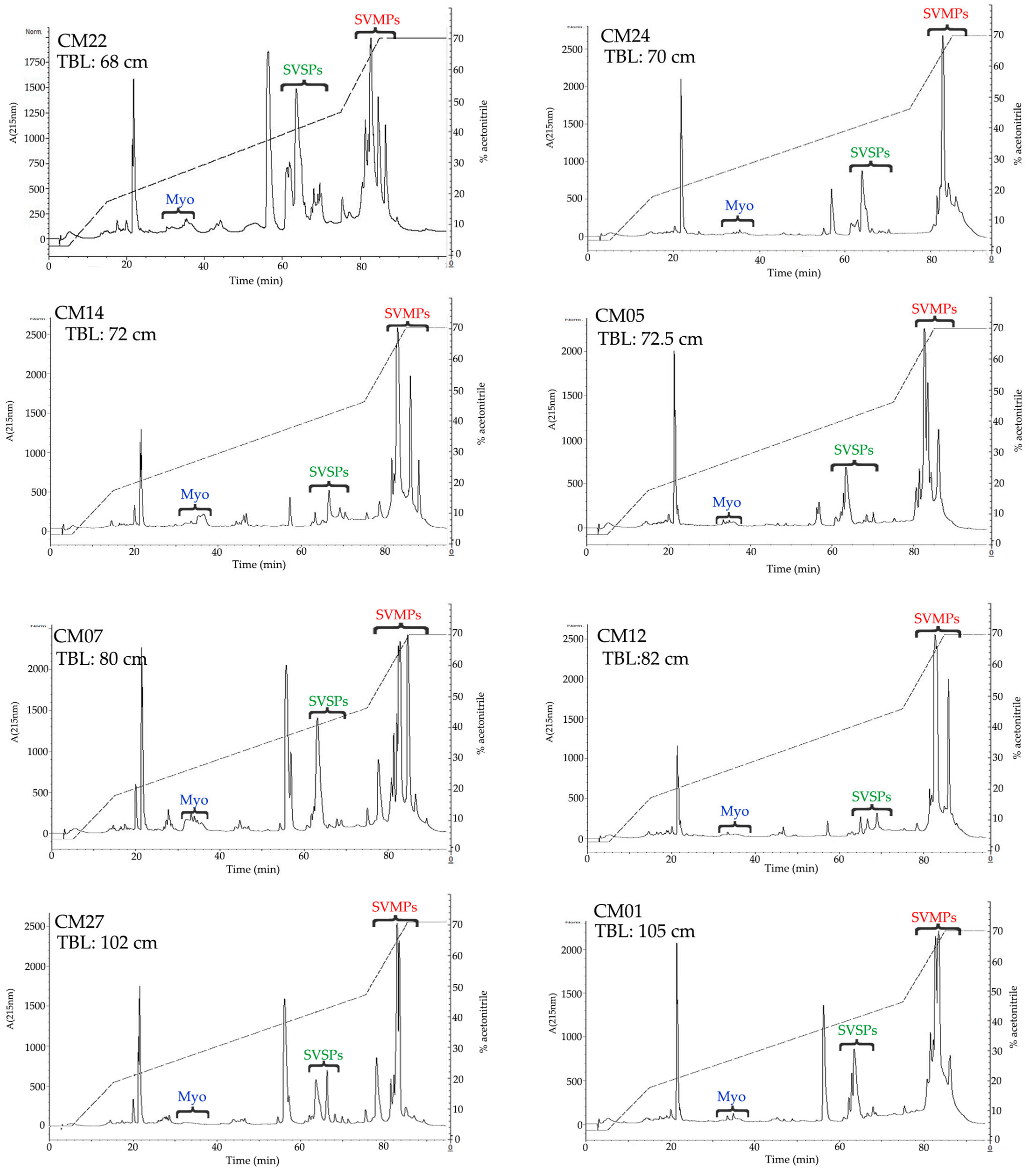

Figure S1. Reverse-phase HPLC chromatograms of remaining *C. m. nigrescens* venoms of snakes with different total body length (TBL). An analytic C18 reverse-phase column (Vydac®, Deerfield, IL, USA, 218 TP 4.6 mm × 250 mm) was used. Retention time is along the x axis for each panel and labeled every twenty min. Proteins were detected at 215 nm and absorbance is indicated on the left axis. The acetonitrile gradient is shown in the HPLC graph and the percentage value corresponds to the right axis for each panel. The

regions where crotamine-like myotoxins (Myo), snake venom serine proteases (SVSPs), and snake venom metalloproteinases (SVMPs) elute are illustrated in blue, green and red, respectively.

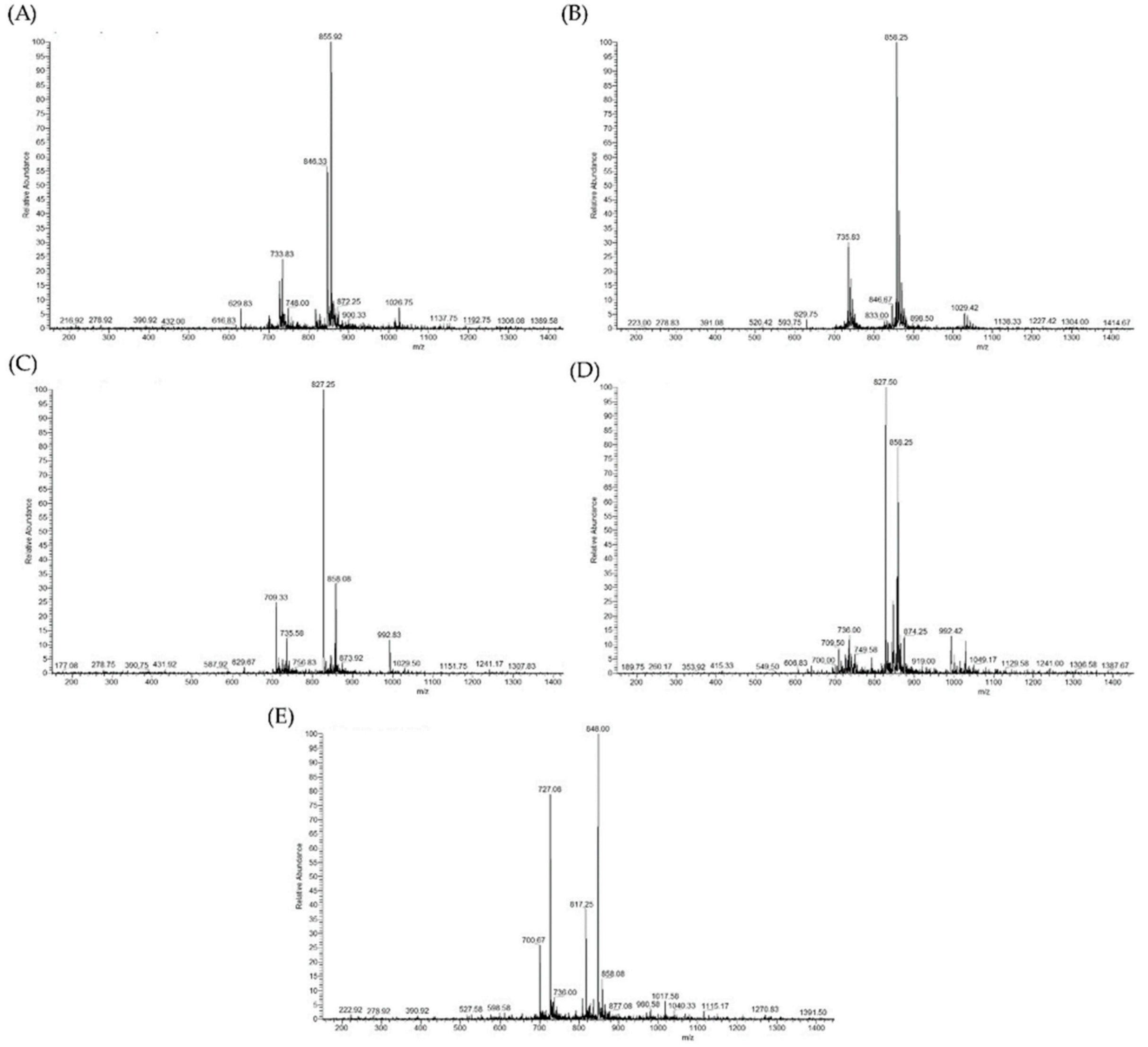

Figure S2. Mass spectrometry of crotamine-like myotoxins. RP-HPLC fractions 6 (**A**), 7 (**B**), 8 (**C**), 9 (**D**) and 10 (**E**) obtained from CM04 venom were analyzed using ESI-MS on an LCQ Fleet Ion Trap Mass Spectrometer. Molecular masses calculated were 5129 Da (**A**), 5071 Da (**A**), 5143 Da (**B**), 5180 Da (**B**), 4958 Da (**C**), 5143 Da (**D**), and 5082 Da (**E**).
